# Association of psychological status and patient-reported physical outcome measures in joint arthroplasty: a lack of divergent validity

**DOI:** 10.1186/1477-7525-11-64

**Published:** 2013-04-19

**Authors:** Johannes M Giesinger, Markus S Kuster, Henrik Behrend, Karlmeinrad Giesinger

**Affiliations:** 1 Department of Psychiatry and Psychotherapy, Innsbruck Medical University, Anichstr, 35, Innsbruck, A-6020, Austria; 2 Department of Orthopaedic Surgery, Royal Perth Hospital, University of Western Australia, Wellington Street, Perth, WA, 6000, Australia; 3 Department of Orthopaedic Surgery, Kantonsspital St. Gallen, Rorschacherstrasse 95, CH- 9000 St., Gallen, Switzerland

## Abstract

**Background:**

Patient-reported outcome measures have become a well-recognised part of outcome assessment in orthopaedic surgery. These questionnaires claim to measure joint-specific dimensions like pain, function in activities of daily living, joint awareness or stiffness. Interference of the patient’s psychological status with these orthopaedic questionnaires however may make accurate interpretation difficult.

**Methods:**

We recruited 356 patients after unilateral, primary THA or TKA and performed a postal survey including the Brief Symptom Inventory (psychological distress measure), the Catastrophising Scale (from the Coping Strategies Questionnaire), the WOMAC score (Western Ontario and McMaster Universities Osteoarthritis Index) and the Forgotten Joint Score – 12 (FJS-12). Associations between the different questionnaires were determined calculating Pearson correlation coefficients. Two multiple linear regression models were used to investigate the impact of socio-demographic variables, clinical variables and of the psychological scales (BSI and Catastrophising Scale) separately for the WOMAC score and the FJS-12.

**Results:**

WOMAC-Total score showed strong correlation to Catastrophising (r = 0.79), BSI-Somatisation (r = 0.63) and BSI-GSI (r = 0.54). The FJS-12 demonstrated modest to strong correlation with Catastrophising (r = −0.60), BSI-Somatisation (r = −0.49) and the BSI-GSI (Global Severity Index) (r = −0.44). BSI-GSI and Catastrophising explained 54.3% of variance in a multivariate regression model for the WOMAC score. The same two scales explained 30.0% of variance for the FJS-12.

**Conclusions:**

There is a strong relationship between psychological status and orthopaedic outcome. The scale names of orthopaedic outcome measures suggest to measure specific dimensions like pain, stiffness, function or joint awareness. In fact they largely include patient’s psychological status indicating poor divergent validity.

## Introduction

There is widespread recognition that assessment of patient outcome following total hip and total knee arthroplasty (THA and TKA respectively) should employ patient-reported outcome (PRO) measures. These tools allow a more patient-centred view in treatment evaluation [1-3] and advocates suggest that they provide a remarkably sophisticated evaluation of whether a treatment has worked in the (important) sense of whether or not the patient feels better, and how much better [4]. Consequently a number of disease and joint-specific PRO assessment instruments have been developed for use with orthopaedic conditions [5-8]. These outcome questionnaires focus mainly on the patients function in typical activities of daily living (ADLs), pain intensity or joint stiffness. They are often employed in tandem with more generic health outcome instruments such as the SF-36 which in addition to assessing physical health incorporates questions on psycho-social aspects of general health. Some generic tools such as the SF-12 have separate summary scores for physical and mental health. Tools such as this have been shown to display good divergent validity [9] in that there is very little interaction between physical and mental component questions and thus overall scores. Interestingly in disease-specific scores that do not have specific mental health components, significant correlation of psychological variables and disease specific variables has been demonstrated [10-12]. This interaction is somewhat expected as poor physical outcome and pain after THA/TKA can cause psychological distress and reduce quality of life, or alternatively, poor psychological status can result in worse physical outcome by interfering with the patients’ compliance to treatment [13] and affect pain coping strategies [14]. Such causal dependency is probably bidirectional with the directions difficult to separate. An alternative explanation though to the overlap in mental and physical health parameters in these assessment tools is a failure of the patient-reported outcome measure to discriminate the overlapping constructs, and thus poor divergent validity [15,16]. A lack of divergent validity means that interpretability of such scales is limited since the resulting scores blend different constructs. Poor outcome scores can then reflect poor physical outcome, poor psychological status, or both. It is clearly desirable to use a diagnostic tool that separates physical from psychological variables as well as possible if one wishes to assess physical function in isolation.

Thresholds for correlations as indicators of divergent validity are rarely explicitly stated in the literature. However, some studies suggest that correlations below 0.30 indicate divergent validity [17,18], whereas correlations above 0.40 are considered as indicating convergent validity [19].

The aim of this study was to evaluate the divergent validity of the WOMAC score and the Forgotten Joint Score, and to investigate correlations with psychological variables after joint arthroplasty.

## Patients and methods

### Sample

All patients that underwent THA or TKA at our institution within the last five years were considered for enrolment in this study and approached for study participation at their follow-up visits in 2008.

Inclusion criteria were: unilateral THA (cemented Stuemer-Weber hip stem, uncemented Fitmore cup, Zimmer) or unilateral TKA (cemented LCS complete, DePuy), primary arthroplasty surgery, no previous THA or TKA surgery.

Sociodemographic and clinical data including sex, age, education, type and location of implant and time since surgery were collected. Patients were sent the questionnaires and an informed consent form via mail. A reminder call was made to those patients who did not send back the questionnaires within eight weeks. If there was no response for another four weeks they were excluded. Reasons for not participating in the study were recorded.

Ethical approval for this study was obtained from the ethics committee of the canton of St Gallen, Switzerland.

### Assessment instruments

#### Forgotten joint score-12

The Forgotten Joint Score-12 (FJS-12) is a recently published PRO measure to assess joint awareness in hips and knees during various activities of daily living [6]. It consists of 12 questions and is scored using a 5-point Likert response format with the raw scores transformed onto a 0–100 point scale. High scores indicate good outcome. The FJS has been shown to have a low ceiling effect and discriminates well between good, very good and excellent outcome after THA and TKA. It has shown high internal consistency (Cronbach’s Alpha 0.95) and discriminates well in known group comparisons [6].

#### Western Ontario and McMaster Universities Osteoarthritis Index

The Western Ontario and McMaster Universities (WOMAC) Osteoarthritis Index is a widely used outcome measure in patients with lower limb osteoarthritis (OA) [5]. It consists of 24 questions covering three dimensions: pain (five questions), stiffness (two questions) and function (17 questions). Scale scores are derived from adding up the item scores. High scores indicate poor outcome. The WOMAC OA index has been extensively tested for validity, reliability, feasibility and responsiveness for measuring changes after different OA interventions [5,20-22].

#### Brief symptom inventory

The Brief Symptom Inventory (BSI) [23] is a psychological self-report symptom scale developed as a short-form version of the SCL-90-R [24]. It is widely used in various medical fields to assess current psychological status and distress. The 53 items are grouped in nine symptom scales (somatisation, obsessive-compulsive behaviour, interpersonal sensitivity, depression, anxiety, hostility, phobic anxiety, paranoid ideation, and psychoticism) and three global indices, Global Severity Index (GSI) as a global distress measure, Positive Symptom Distress Index (PSDI), and Positive Symptom Total (PST). Scale scores are derived from mean item scores. High scores indicate high psychological symptom burden.

#### Catastrophising scale

The catastrophising scale is part of the Coping Strategies Questionnaire developed by Rosenstiel and Keefe [25]. It comprises six items assessing catastrophising as a pain-related coping strategy characterised by a feeling of being overstrained and a pessimistic future perspective. The scale scores are derived from adding up the items. A high score indicates poor coping.

### Statistical analysis

Sample characteristics are presented as percentages or as means with standard deviations and ranges. For determining associations between the administered scales (WOMAC score, FJS-12, BSI, Catastrophising scale) Pearson-correlation coefficients were calculated. Two multiple linear regression models were used to investigate the impact of sociodemographic and clinical variables and of the psychological scales (BSI and Catastrophising scale) separately for the WOMAC and for the FJS-12 score. In these models adjusted R-Squared (R^2^) indicates the proportion of variance explained by the independent variables (predictors) in the model. Variables having a significant association with the WOMAC or the FJS-12 in univariate analyis were considered for inclusion into the multivariate regression model if p < 0.05. In a first block of predictors, the patient characteristics sex, education, and location were included. In a second block of predictors the psychological scales (BSI scales and the Catastrophising scale) were included using a forward selection procedure.

## Results

### Sample characteristics

A total number of 356 patients were contacted in a mail survey in August 2008. 243 (68.3%) patients returned the questionnaires along with written informed consent. Reasons for not participating in the study (phone call) were: refusal of participation (42 patients; 11.8%), wrong address (29 patients; 8.1%), death (22 patients; 6.2%), cognitive impairment (3 patients; 0.8%), moving abroad (1 patient; 0.3%) and unknown reasons (16 patients; 4.5%). Mean patient age was 70.6 (SD 11.3) and 120 patients (49.4%) were female. 157 (64.6%) patients had THA surgery and 86 (35.4%) had TKA surgery. For further details see Table 1.

**Table 1:** Descriptive statistics for clinical and socio-demographic variables (n = 243)

Gender	Male	123/243 (50.6%)
	Female	120/243 (49.4%)
Age	Mean (SD)	70.6 (11.3)
	Range	32-91
Education	Compulsory school	54/243 (22.2%)
	Apprenticeship	104/243 (42.8%)
	A-level/professional school	39/243 (16.0%)
	University	13/243 (5.3%)
	Missing	33/243 (13.5%)
Location	THA	157/243 (64.6%)
	TKA	86/243 (35.4%)
Side	Left	116/243 (47.7%)
	Right	127/243 (52.3%)
Time since surgery (months)	Mean (SD)	31.1 (12.3)
	Range	15-42

### Correlations between FJS-12, WOMAC, BSI and the catastrophising-scale

Correlation coefficients for the relationship between WOMAC, FJS-12, BSI scales and catastrophising scale are presented in Table 2. Highest correlations for the FJS-12 were found for Catastrophising (r = −0.60), BSI-Somatisation (r = −0.49) and the BSI-GSI (r = −0.44). WOMAC-Total also showed the strongest relation to Catastrophising (r = 0.79), BSI-Somatisation (r = 0.63) and BSI-GSI (r = 0.54). For comparison, correlations between the WOMAC subscales (pain, stiffness, and function) were between r = 0.80 and r = 0.91.

**Table 2:** Correlations between WOMAC, FJS-12, Catastrophising and BSI

	**FJS-12**	**WOMAC Total**	**WOMAC Pain**	**WOMAC Stiffness**	**WOMAC Function**
Catastrophising	−0.60	0.79	0.78	0.60	0.77
BSI Somatisation	−0.49	0.63	0.60	0.53	0.64
BSI Obsessive-compulsive	−0.33	0.39	0.36	0.36	0.39
BSI Interpersonal sensitivity	−0.34	0.39	0.38	0.30	0.39
BSI Depression	−0.28	0.39	0.37	0.28	0.39
BSI Anxiety	−0.38	0.49	0.47	0.40	0.50
BSI Hostility	−0.33	0.38	0.37	0.26	0.38
BSI Phobic anxiety	−0.39	0.46	0.44	0.42	0.45
BSI Paranoid ideation	−0.32	0.41	0.41	0.25	0.40
BSI Psychoticism	−0.30	0.35	0.34	0.28	0.35
BSI GSI	−0.44	0.54	0.52	0.43	0.54

All correlations are significant at the 0.01 level (two-tailed). Negative correlations reflect the direction of the scoring used for the FJS-12.

### Multivariate analysis of the FJS-12 and the WOMAC score

Sex, education and location of implant (hip or knee) have previously been shown to impact on the FJS-12 and WOMAC-Total score [6]. These variables were included as predictors in two separate linear regression models, with the WOMAC total score and FJS-12 as the dependant variables. The global distress scale of the BSI (BSI-GSI) as well as BSI-Somatisation and the Catastrophising scale were included as predictors in both models.

**Figure 1:**
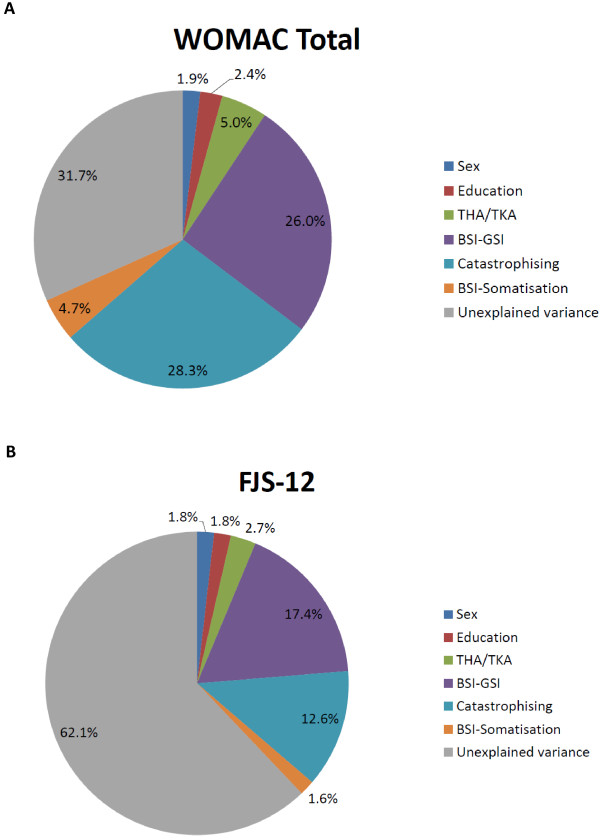
Explained and unexplained variance for the WOMAC (1a) and FJS-12 scores (1b).

Overall the demographic and psychological variables explained 38% of the variance in the FJS-12 and 68% of the variance in the WOMAC score. Gender, education, and implant location (hip or knee replacement) explained similar small proportions of each score (gender explained 1.8% of the variance in FJS-12 and 1.9% of WOMAC-Total score; Education 1.8% of FJS-12 and 2.4% of WOMAC-Total score; and implant location, 2.7% of the FJS-12 and 5.0% of the WOMAC-Total score). Larger discrepancies were seen between WOMAC and FJS-12 in terms of the amount of variance explained by BSI-GSI scale (17.4% of FJS-12, and 26.0% of WOMAC-Total score), Catastrophising scale (12.6% of FJS-12, and 28.3% of WOMAC-Total score), and the BSI-Somatisation scale (1.6% of FJS-12, and 4.7% of WOMAC-Total score) (Table 3 and Figure 1).

**Table 3:** Multiple linear regression model for FJS-12 and WOMAC-Total

	**FJS-12**				** WOMAC-Total**			
**Predictors**	**Adjusted R**^**2**^	**Change adjusted R**^**2**^	**F**	**p**	**Adjusted R**^**2**^	**Change adjusted R**^**2**^	**F**	**p**
Gender	0.018	0.018	4.75	0.030	0.019	0.019	4.84	0.029
+ Education	0.036	0.018	2.88	0.024	0.043	0.024	2.18	0.014
+ Location	0.063	0.027	3.67	0.003	0.093	0.050	3.24	<0.001
+ BSI-GSI	0.237	0.174	11.34	<0.001	0.353	0.260	8.71	<0.001
+ Catastrophising	0.363	0.126	17.29	<0.001	0.636	0.283	13.00	<0.001
+ BSI-Somatisation	0.379	0.016	16.27	<0.001	0.683	0.047	12.20	<0.001

Equations for the final regression models (unstandardised):

WOMAC Total = −5.176 + 0.986*sex - 1.614*education_d1 - 3.503*education_d2 -3.939*education_d3 + 2.058*location + 0.311*BSI-GSI + 7.984*Catastrophising + 13.292*BSI-Somatisation.

FJS-12 = 84.521 - 2.258*sex + 0.540*education_d1 + 3.125*education_d2 + 13.073*education_d3 - 4.178*location - 7.105*BSI-GSI - 8.675*Catastrophising - 13.102*BSI-Somatisation.

Coding of predictors:

Sex: Male = 0, Female = 1.

Education (dummy-coded):

Apprenticeship: d1 = 1.

A-level/professional school: d2 = 1.

University: d3 = 1.

Else: d1. d2. d3 = 0.

Location: 1 = THA, 2 = TKA.

## Discussion

This study investigated the associations between psychological parameters and physical outcome assessed by two PRO instruments, the WOMAC score and the FJS-12. We found high correlations between disease-specific outcome measures and several of the assessed psychological domains. Multivariate regression showed that catastrophising, psychological distress and somatisation explained almost 60% variance of the WOMAC score beyond the known covariates of sex, implant location and education. We found the same predictor set for the FJS-12, however, psychological parameters accounted only for half the variance seen in the WOMAC score.

Our findings indicate a significant lack of divergent validity of the WOMAC score and, to a lesser extent, of the FJS-12. The variance proportions estimated with help of the regression model suggest a substantial overlap between the orthopaedic and psychological scales. The lack of divergent validity becomes even more evident when opposing the high correlations between the WOMAC subscales themselves (above 0.80) and the correlations of the WOMAC total score with the psychological scores (up to 0.79).

This significant overlap with psychological status is not reflected in the WOMAC scales’ names (pain, stiffness, function) which somewhat misleadingly suggest to just measure physical, joint-related characteristics. This is also true for the FJS-12 which refers to joint awareness. However, the term joint awareness seems more closely related to psychological aspects.

We also found that location of joint arthroplasty (hip or knee) explained less than 5% of variance of both FJS-12 and the WOMAC score. This is interesting as it is well accepted that outcome differs between total hip and total knee arthroplasty populations [26,27]. In contrast, the psychological scales exceeded these proportions by a factor of 10 (for both FJS-12 and WOMAC). Thus, our data indicate a stronger association between psychological factors and joint-related outcomes than that between outcome and the type of joint replaced.

Our findings compare well to other results from literature. Escobar et al. [15] investigated the association between WOMAC scores and the different subscales of the SF-36. They showed that both psycho-social and physical SF-36 scales correlated to the WOMAC score in a similar way. The WOMAC function subscale demonstrated the same correlation with both SF-36 social and physical function scores. WOMAC stiffness was equally correlated with SF-36 role-physical function score and mental health score. Similarly Wolfe [16] highlighted that divergent validity of the WOMAC may be compromised by factors such as fatigue, symptom counts, depression, and low back pain.

The strong correlation between physical and psychological scales found here and in other studies [28-30] may partially be explained by causal interdepencies that have been suggested by several longitudinal studies.

Sharma et al. [31] demonstrated that mental health measured with the SF-36 predicted subsequent improvement in physical function in TKA, results in line with Brander et al. [32], who showed that preoperative depression substantially influences Knee Society Rating Scale function scores five years post-operatively. In contrast, Lingard et al. [33] found (in a large prospective observational study) that although psychological distress decreased post-operatively, pre-operative levels of distress were not related to post-operative improvement (change in pain and function).

Lopez-Olivo et al. [12] found a strong correlation between pre-operative psychological status and post-operative physical function at 6 months. Education, coping style and locus of control over health at baseline explained 22% of variance in WOMAC pain at follow-up. A similar predictor-set explained 19% of the WOMAC function scale and 36% of the total score of the Knee Society Rating Scale.

Our study was based on a cross-sectional design which is reasonable for the investigation of divergent validity. However, it does not allow for causal interpretation of the associations between orthopaedic outcomes and psychological variables. A limitation is the limited number of predictors in our model that left a large proportion of unexplained variance. Further interesting predictors that may be of future research interest include patient activity level, social support, cognitive function, range of motion and joint stability.

A particular strength of this study is the use of a comprehensive and detailed assessment of psychological status (BSI and the Catastrophising Scale from the Coping Strategies Questionnaire). These scales are more differentiated and comprehensive than other tools such as the SF-36 which has previously been employed to assess psychosocial characteristics of arthroplasty populations.

## Conclusion

We found a substantial overlap between physical and psychological patient-reported symptoms in an arthroplasty population, i.e. orthopaedic PRO measures were strongly associated with psychological PRO measures indicating poor divergent validity.Whereas this may also reflect existing causal dependencies, it impairs valid measurement of orthopaedic outcome. Divergent validity is an important psychometric characteristic of PRO instruments that is required to guarantee accurate assessment of specific orthopaedic outcomes.

Problematically, the category names of the orthopaedic outcome scales suggest measurement of specific constructs such as pain, stiffness, function or joint awareness but they appear to be strongly associated with patients’ psychological status. Our findings suggest that the names of certain orthopaedic scales do not adequately reflect the constructs assessed with these scales.

## Competing interests

The authors declare that they have no competing interests.

## Authors’ contributions

KG, MSK and JMG conceived the study objective. All authors participated in the study design. KG and HB coordinated data collection. JMG and KG performed the statistical analysis, interpreted the results and drafted the manuscript. All authors read and approved the final manuscript.

## References

[B1] Wright RW Knee injury outcomes measures J Am Acad Orthop Surg 2009 17 31 39 19136425 10.5435/00124635-200901000-00005

[B2] Suk M Norvell DC Hanson B Dettori JR Helfet D Evidence-based orthopaedic surgery: what is evidence without the outcomes? J Am Acad Orthop Surg 2008 16 123 129 18316710 10.5435/00124635-200803000-00003

[B3] Pollard B Johnston M Dixon D Theoretical framework and methodological development of common subjective health outcome measures in osteoarthritis: a critical review Health Qual Life Outcomes 2007 5 14 10.1186/1477-7525-5-14 17343739 PMC1832179

[B4] Timmins N NHS goes to the PROMS BMJ 2008 336 1464 1465 10.1136/bmj.39618.627951.80 18583674 PMC2440853

[B5] Bellamy N Buchanan WW Goldsmith CH Campbell J Stitt LW Validation study of WOMAC: a health status instrument for measuring clinically important patient relevant outcomes to antirheumatic drug therapy in patients with osteoarthritis of the hip or knee J Rheumatol 1988 15 1833 1840 3068365

[B6] Behrend H Giesinger K Giesinger JM Kuster MS The “Forgotten Joint” as the Ultimate Goal in Joint Arthroplasty Validation of a New Patient-Reported Outcome Measure J Arthroplasty 2012 27 430 436 10.1016/j.arth.2011.06.035 22000572

[B7] Dawson J Fitzpatrick R Carr A Murray D Questionnaire on the perceptions of patients about total hip replacement J Bone Joint Surg Br 1996 78 185 190 8666621

[B8] Dawson J Fitzpatrick R Murray D Carr A Questionnaire on the perceptions of patients about total knee replacement J Bone Joint Surg Br 1998 80 63 69 10.1302/0301-620X.80B1.7859 9460955

[B9] Ware J Kosinski M Keller S SF-12: How to Score the Sf-12 Physical and Mental Health Summary Scales 1998 3 Lincoln, RI: QualityMetric Incorporated

[B10] Axford J Butt A Heron C Hammond J Morgan J Alavi A Bolton J Bland M Prevalence of anxiety and depression in osteoarthritis: use of the Hospital Anxiety and Depression Scale as a screening tool Clin Rheumatol 2010 29 1277 1283 10.1007/s10067-010-1547-7 20721594

[B11] Lavernia CJ Alcerro JC Brooks LG Rossi MD Mental health and outcomes in primary total joint arthroplasty J Arthroplasty 2012 27 7 1276 1282 10.1016/j.arth.2011.11.015 22226610

[B12] Lopez-Olivo MA Landon GC Siff SJ Edelstein D Pak C Kallen MA Stanley M Zhang H Robinson KC Suarez-Almazor ME Psychosocial determinants of outcomes in knee replacement Ann Rheum Dis 2011 70 1775 1781 10.1136/ard.2010.146423 21791452

[B13] Lenze EJ Munin MC Dew MA Rogers JC Seligman K Mulsant BH Reynolds CF 3rd Adverse effects of depression and cognitive impairment on rehabilitation participation and recovery from hip fracture Int J Geriatr Psychiatry 2004 19 472 478 10.1002/gps.1116 15156549

[B14] Somers TJ Keefe FJ Pells JJ Dixon KE Waters SJ Riordan PA Blumenthal JA McKee DC LaCaille L Tucker JM Pain catastrophizing and pain-related fear in osteoarthritis patients: relationships to pain and disability J Pain Symptom Manage 2009 37 863 872 10.1016/j.jpainsymman.2008.05.009 19041218 PMC2702756

[B15] Escobar A Quintana JM Bilbao A Azkarate J Guenaga JI Validation of the Spanish version of the WOMAC questionnaire for patients with hip or knee osteoarthritis. Western Ontario and McMaster Universities Osteoarthritis Index Clin Rheumatol 2002 21 466 471 10.1007/s100670200117 12447629

[B16] Wolfe F Determinants of WOMAC function, pain and stiffness scores: evidence for the role of low back pain, symptom counts, fatigue and depression in osteoarthritis, rheumatoid arthritis and fibromyalgia Rheumatology (Oxford) 1999 38 355 361 10.1093/rheumatology/38.4.355 10378714

[B17] de Groot IB Favejee MM Reijman M Verhaar JA Terwee CB The Dutch version of the Knee Injury and Osteoarthritis Outcome Score: a validation study Health Qual Life Outcomes 2008 6 16 10.1186/1477-7525-6-16 18302729 PMC2289810

[B18] Innes E Straker L Validity of work-related assessments Work 1999 13 125 152 12441557

[B19] Fayers P Machin D Quality of Life: The Assessment, Analysis and Interpretation of Patient-reported Outcomes 2007 2 Chichester, UK: John Wiley & Sons

[B20] Impellizzeri FM Mannion AF Leunig M Bizzini M Naal FD Comparison of the reliability, responsiveness, and construct validity of 4 different questionnaires for evaluating outcomes after total knee arthroplasty J Arthroplasty 2011 26 861 869 10.1016/j.arth.2010.07.027 21074964

[B21] Wolfe F Kong SX Rasch analysis of the Western Ontario MacMaster questionnaire (WOMAC) in 2205 patients with osteoarthritis, rheumatoid arthritis, and fibromyalgia Ann Rheum Dis 1999 58 563 568 10.1136/ard.58.9.563 10460190 PMC1752940

[B22] Terwee CB Roorda LD Knol DL De Boer MR De Vet HC Linking measurement error to minimal important change of patient-reported outcomes J Clin Epidemiol 2009 62 1062 1067 10.1016/j.jclinepi.2008.10.011 19230609

[B23] Derogatis LR BSI Brief Symptom Inventory: Administration, Scoring and Procedures Manual (4th edition) 1993 National Computer Systems: Minneapolis, MN

[B24] Derogatis LR SCL-90-R Revised Manual 1983 Baltimore: John Hopkins School of Medicine

[B25] Rosenstiel AK Keefe FJ The use of coping strategies in chronic low back pain patients: relationship to patient characteristics and current adjustment Pain 1983 17 33 44 10.1016/0304-3959(83)90125-2 6226916

[B26] Bourne RB Chesworth B Davis A Mahomed N Charron K Comparing patient outcomes after THA and TKA: is there a difference? Clin Orthop Relat Res 2010 468 542 546 10.1007/s11999-009-1046-9 19760472 PMC2806999

[B27] Hamilton D Henderson GR Gaston P Macdonald D Howie C Simpson AH Comparative outcomes of total hip and knee arthroplasty: a prospective cohort study Postgrad Med J 2012 88 627 631 10.1136/postgradmedj-2011-130715 22822221

[B28] Pallant JF Keenan AM Misajon R Conaghan PG Tennant A Measuring the impact and distress of osteoarthritis from the patients’ perspective Health Qual Life Outcomes 2009 7 37 10.1186/1477-7525-7-37 19400966 PMC2683800

[B29] van Baar ME Dekker J Lemmens JA Oostendorp RA Bijlsma JW Pain and disability in patients with osteoarthritis of hip or knee: the relationship with articular, kinesiological, and psychological characteristics J Rheumatol 1998 25 125 133 9458215

[B30] Salaffi F Cavalieri F Nolli M Ferraccioli G Analysis of disability in knee osteoarthritis. Relationship with age and psychological variables but not with radiographic score J Rheumatol 1991 18 1581 1586 1765985

[B31] Sharma L Cahue S Song J Hayes K Pai YC Dunlop D Physical functioning over three years in knee osteoarthritis: role of psychosocial, local mechanical, and neuromuscular factors Arthritis Rheum 2003 48 3359 3370 10.1002/art.11420 14673987

[B32] Brander V Gondek S Martin E Stulberg SD Pain and depression influence outcome 5 years after knee replacement surgery Clin Orthop Relat Res 2007 464 21 26 17603386 10.1097/BLO.0b013e318126c032

[B33] Lingard EA Riddle DL Impact of psychological distress on pain and function following knee arthroplasty J Bone Joint Surg Am 2007 89 1161 1169 10.2106/JBJS.F.00914 17545417

